# Combination of circulating tumor cells and 18F‐FDG PET/CT for precision diagnosis in patients with non‐small cell lung cancer

**DOI:** 10.1002/cam4.70216

**Published:** 2024-09-20

**Authors:** Momo Sun, Dongyan Lu, Xiaoping Li, Jin Wang, Liang Zhang, Pan Yang, Yang Yang, Jie Shen

**Affiliations:** ^1^ The First Central Clinical School Tianjin Medical University Tianjin China; ^2^ Department of Nuclear Medicine Tianjin First Central Hospital Tianjin China; ^3^ Department of Thoracic Surgery Tianjin First Central Hospital Tianjin China; ^4^ Nankai University Tianjin China

**Keywords:** 18F‐FDG PET/CT, circulating tumor cells (CTCs), diagnosis, non‐small‐cell lung cancer (NSCLC)

## Abstract

**Purpose:**

To investigate the value of 2‐deoxy‐18f‐fluorodeoxyglucose (18F‐FDG) positron emission tomography/computed tomography (PET/CT) and circulating tumor cells (CTCs) for the differential diagnosis of patients with benign lung diseases and those with NSCLC. To explore the phenotypic heterogeneity of CTCs and their correlation with FDG uptake in patients with Stage I–IV NSCLC.

**Methods:**

Blood specimens from patients with benign lung diseases and patients with primary NSCLC were collected for the detection of CTCs and their subtypes (epithelial, mixed, and mesenchymal) and analyzed for 18F‐FDG PET/CT tumor metabolic parameters, including the maximum standardized uptake value (SUV_max_), standard uptake value (SUL), metabolic tumor volume of primary lesion (MTV), total lesion glycolysis of primary lesion (TLG). Clinical data including age, gender, smoking history, tumor size, TNM stage and pathology type were also collected. The value of the two method alone and in combination for the differential diagnosis of benign and malignant was comparatively analyzed. Finally, the differences in CTC and its subtypes in different stages of NSCLC were compared, and FDG metabolic parameters were correlated with CTC subtypes.

**Results:**

There were a total of 65 patients with pulmonary diseases, including 12 patients with benign pulmonary diseases and 53 patients with NSCLC. The mean age was 67 ± 10 (38–89 years), 27 were females and 38 were males. 31 (22 males and 9 females) had a long history of smoking. The mean size of the largest diameter of all single lesions was 36 ± 22 mm with a range of 10–108 mm. Seven out of 12 benign diseases were inflammatory granulomatous lesions and 5 were inflammatory pseudotumours. Twenty‐four out of 53 NSCLC were adenocarcinomas and 29 were squamous carcinomas. Twelve out of 53 patients with NSCLC were in Stage I, 10 were in Stage II, 17 were in Stage III and 14 were in Stage IV. SUV_max_, SUL, MTV, TLG, total CTCs, epithelial CTCs, and mixed CTCs were all valuable in the differential diagnosis of benign and malignant. TLG combined with mixed CTCs was statistically different from all other diagnostic methods (*p* < 0.05) and higher than any other diagnostic criteria. In the differential diagnosis of benign and Stage I NSCLC, only total CTC (*Z* = −2.188 *p* = 0.039) and mixed CTCs (*Z* = −3.020 *p* = 0.014) had certain diagnostic efficacy, and there was no statistical difference between them (*p* = 0.480). Only mesenchymal CTCs differed in Stage I–IV NSCLC, with a higher number of those who developed distant metastases than those who had non‐distant metastases. Epithelial CTCs correlated with SUV_max_ (*r* = 0.333, *p* = 0.015) and SUL (*r* = 0.374, *p* = 0.006). Mmesenchymal CTCs correlated with MTV (*r* = 0.342, *p* = 0.018) and TLG (*r* = 0.319, *p* = 0.02). Further subgroup analyses revealed epithelial CTCs were correlated with SUV_max_ (*r* = 0.543, *p* = 0.009) and SUL (*r* = 0.552, *p* = 0.008), and the total CTCs was correlated with SUV_max_ (*r* = 0.622, *p* = 0.003), SUL (*r* = 0.652, *p* = 0.003), MTV (*r* = 0.460, *p* = 0.031), and TLG (*r* = 0.472, *p* = 0.027) in the early group (Stage I–II). Only mesenchymal CTCs was associated with MTV (*r* = 0.369, *p* = 0.041), and TLG (*r* = 0.415, *p* = 0.02) in the intermediate‐late group (Stage III–IV).

**Conclusion:**

Both FDG PET metabolic parameters and CTCs demonstrated diagnostic value for NSCLC, and combining TLG with mixed CTCs could enhance their diagnostic efficacy. The total CTCs and mixed CTCs showed greater diagnostic value than FDG PET in distinguishing benign lesions from Stage I NSCLC. In NSCLC patients, the epithelial CTCs exhibited a positive correlation with SUV_max_ and SUL, while mesenchymal CTCs correlated with MTV, and TLG. Besides, epithelial CTCs showed stronger correlations with SUV_max_ and SUL, and total CTCs showed stronger correlations with SUV_max_, SUL, MTV, and TLG in Stage I–II NSCLC. Only mesenchymal CTCs in Stage III–IV NSCLC showed correlations with MTV and TLG. Stage IV NSCLC cases displayed a higher number of mesenchymal CTCs.

## 1 BACKGROUND

According to the “Cancer Statistics 2023” report,^1^ lung cancer accounts for approximately 12.5% of new cancer cases globally, making it the second most common and deadliest cancer type. NSCLC constitutes the majority of lung cancer cases, representing 85% of new diagnoses.^2^ Despite efforts to improve the diagnosis and treatment of NSCLC, the five‐year survival rate remains notably low. Research indicates that while some early‐stage patients can undergo surgery, 30%–55% of patients experience recurrence and distant metastasis, leading to a mortality rate of up to 50%.^3^ This suggests the potential for early‐stage spread of cancer cells. Additionally, many patients are diagnosed with advanced‐stage disease, resulting in lower rates of progression‐free survival (PFS) and overall survival (OS) in this subgroup.^4^ Therefore, early diagnosis of NSCLC and understanding its development and spread mechanisms are crucial areas of focus.

By providing semi‐quantitative parameters of tumor glucose metabolism, 18f‐fluorodeoxyglucose (18F‐FDG) positron emission tomography/computed tomography (PET/CT) can supply additional information on the diagnosis, staging, and prognosis of NSCLC.^5^ Although several studies^6^, ^7^, ^8^ have linked the intensity of FDG uptake to tumor metastatic potential, the mechanism of this association remains unclear. In addition to imaging, the detection of circulating tumor cells (CTCs) in peripheral blood has been investigated as an evaluative biomarker for NSCLC. This approach, which is noninvasive and easily reproducible, allows dynamic disease monitoring and offers valuable information on tumor heterogeneity and the biological mechanisms driving metastasis‐initiating cells. Nevertheless, detection technology for CTCs remains quite challenging. Several studies^9^, ^10^ have confirmed the close relationship between epithelial–mesenchymal transition (EMT) and reprogrammed cancer metabolism at the molecular biological level. As tumor glucose metabolism is influenced by the Warburg effect, leading to increased FDG uptake in PET scans, the number and isoforms of CTCs also change correspondingly. However, the relationship between CTCs and tumor glucose metabolism is still relatively unexplored in clinical practice.

Given the respective limitations and potential relevance between FDG PET and CTCs, the first objective of this study was to investigate whether the combined assessment of PET‐CT and CTCs could improve the diagnostic efficacy of NSCLC. The secondary objective was to examine the correlation between the phenotypic heterogeneity of CTCs and FDG uptake in NSCLC patients and to determine whether CTCs could provide more prognostic information than PET‐CT.

## 2 METHODS

### 2.1 Patient population

This was a single‐center, prospective, observational study involving patients with treatment‐naive NSCLC and benign pulmonary lesions (including inflammatory lesions and pulmonary tuberculosis) who underwent 18F‐FDG PET/CT examination and peripheral blood CTCs analysis. The inclusion criteria were as follows: (1) no history of prior systemic or local treatment; (2) absence of concurrent malignancies or cancer history; and (3) subsequent pathological examination for lung lesions. The exclusion criteria included (1) a history of other types of cancer, (2) blood sample coagulation, (3) blood sample hemolysis, and (4) blood sample volume less than 5 mL. All procedures involving human subjects in this research were approved by the Ethics Committee of Tianjin First Central Hospital and were conducted in accordance with the principles outlined in the 1964 Declaration of Helsinki and its subsequent amendments or similar ethical standards, with informed consent obtained from all participants. Peripheral venous blood was collected using a vacuum blood collection tube prior to PET/CT examination on the same day. CTCs enrichment was completed within 24 h postcollection.

### 2.2 18F‐FDG PET/CT scan

The 18F‐FDG PET/CT scan was conducted in accordance with the European Association of Nuclear Medicine (EANM) protocol under strict and standardized conditions. A CT64 machine (Siemens Healthineers, Erlangen, Germany) with an axial field of view of 21.6 cm was used for the PET/CT scans. The radiotracer used was 18F‐FDG, which has a radiochemical purity greater than 95%. Prior to imaging, patients fasted for 6 h (the target blood glucose levels were ≤8 mmol/L for diabetic patients and <7 mmol/L for nondiabetic patients receiving 18F‐FDG administration). Intravenous administration of 0.12 mCi 18F‐FDG per kilogram of body weight was performed, followed by image acquisition approximately 60 min later. Each patient underwent 6–7 bed position scans from the head to the mid‐thigh region, with a scan time of 2.5 min per bed position. CT scans were performed on the same scanner without the need for oral or intravenous contrast agents. The CT images were used to correct the PET scans for attenuation with the following scan parameters: 120 kV, 101 mA, gantry rotation time of 0.5 s, and all CT images were acquired with a slice thickness of 5 mm. PET images were reconstructed using iterative reconstruction with a matrix size of 200 × 200 pixels. Finally, the PET and CT images were transferred to a workstation for fusion analysis.

### 2.3 PET/CT imaging analysis

PET/CT images were interpreted by two experienced nuclear medicine physicians who were unaware of the clinical information and pathology findings of the patient. In cases where discrepancies arise from the PET/CT results, a consensus is reached through mutual discussion between them. PET metabolic parameters, including the maximum standardized uptake value (SUV_max_), SUV_mean_, SUV_peak_, SUV corrected by lean body mass (standard uptake value [SUL]), metabolic tumor volume of the primary lesion (MTV), and total lesion glycolysis of the primary lesion (TLG), were analyzed using TrueD software. TLG = SUV_mean_ × MTV.

### 2.4 CTC isolation and enumeration

We used multiple probe‐based immunofluorescence morphology to quantify CTCs and performed subtyping. Peripheral blood samples of 5 mL were collected using an EDTA anticoagulant tube, and the blood specimens were processed within 4 h. First, the blood sample was transferred to a cell preservation tube for lysis of red blood cells. After incubation at room temperature for 30 min, the supernatant was removed by centrifugation at 600 × *g* for 5 min, and the pellet was filtered through an 8 μm pore size nanomembrane. After enrichment of the nanomembrane, the cells were subjected to RNA fluorescence in situ hybridization using six probes (CK8/18/19/Epcam/Vimentin/CD45) at 40°C. Subsequently, preamplification, amplification, and signal hybridization reactions were performed at 40°C to achieve multilevel amplification of the fluorescence signal. Finally, after DAPI staining, the images were scanned, and the results were interpreted and statistically analyzed under an automated fluorescence microscope. The criteria for CTCs determination were as follows: clear nuclear morphology with distinct uneven nuclear staining patterns observed after DAPI staining. Based on the expression of signal points, CTCs are classified as epithelial (CK8/18/19/Epcam^+^, vimentin^−^, CD45^−^), mixed (CK8/18/19/Epcam/Vimentin^+^, CD45^−^), or mesenchymal (Vimentin^+^, CK8/18/19/Epcam^−^, CD45^−^).

### 2.5 Statistical analysis

The data collected in this study were processed and analyzed using SPSS 27.0 software. Count data are expressed as the number of cases (*n*), and categorical data are expressed as the number of cases and percentage (%). Continuous variables were assessed for normality using the Shapiro–Wilk test. Variables conforming to a normal distribution are described using the mean ± standard deviation, while those not conforming to a normal distribution are expressed as the median (quartile). (1) Group comparisons for normally distributed data were performed using an independent samples *t*‐test, and for non‐normally distributed data, the nonparametric rank‐sum test was used. Categorical variables were compared using the chi‐square test or Fisher's exact test; (2) SUV_max_ ≥2.5 and total CTCs ≥1 per 5 mL of peripheral blood were criteria for benign versus malignant determination, with sensitivity and specificity accuracy calculated using the *χ*2 test on a four‐cell scale; (4) GRAPHPAD PRISM 9.1 was used for ROC curve analysis to evaluate various clinical indicators, CTC number and subtypes, and metabolic parameters. The Delong test was used for comparison of the diagnostic efficacy of different ROC curves. (4) Spearman rank correlation analysis was performed for ordered multicategorical variables of the two groups, and correlation curves and heatmaps were generated using GraphPad Prism 9.1 software. *p* < 0.05 were considered to indicate statistical significance.

The SUV threshold of 2.5 is generally used as a cutoff value for distinguishing between lung malignancies and benign diseases, which is based on the results of several previous studies^11^, ^12^, ^13^ that have been published. There is currently no consensus over the optimal cut‐off value of CTCs in the diganosis of NSCLC.The differences may be due to varying CTC enrichment platforms, different blood sample collection times, and differences in patient populations. In the current study, CTC ≥ 1/5 mL was considered positive, irrespective of the CTC subtype. This approach was informed by a previous study^14^ utilizing an identical CTC detection platform and a comparable patient population. In that study, CTC ≥ 0.5/5 mL was identified as the optimal threshold for diagnosing NSCLC.

## 3 RESULTS

### 3.1 Patient characteristics

A total of 70 patients who were initially diagnosed with lung diseases and who underwent whole‐body 18F‐FDG PET/CT and peripheral blood CTC testing from 2022 to 2024 were enrolled in this study; 5 of these patients did not meet the criteria (2 with other types of cancer, 2 with coagulation of blood samples, and 1 with a blood sample size <5 mL). Overall, 65 patients were eligible for further analysis, and the main characteristics of the enrolled patients are shown in Table 1. Twelve patients with pathologically confirmed benign lung disease and 53 patients with NSCLC were enrolled. The mean age was 67 ± 10 years (38–89 years), 27 patients were female, and 38 patients were male. Thirty‐one (22 male and 9 female) patients had a long history of smoking. The mean maximum diameter of all single lesions was 36 ± 22 mm, with a range of 10–108 mm. Out of the 12 benign diseases, 7 were inflammatory granulomatous lesions, and 5 were inflammatory pseudotumors. Out of the 53 NSCLC, 24 were adenocarcinomas, and 29 were squamous carcinomas. TNM staging of the enrolled patients was performed according to IASLC 2023 (TNM staging criteria for lung cancer, 2023, 9th edition). Of the 53 NSCLC patients, 12 had Stage I disease, 10 had Stage II disease, 17 had Stage III disease, and 14 had Stage IV disease.

**TABLE 1:** Patients' characteristics.

Characteristic	Subgroup	*n*	Mean/median	Range
Clinical data				
Total subjects analyzed		65		
Age (years)			67	38–89
Gender	Female	27 (42%)		
	Male	38 (57%)		
Smoking	Smoker	31 (48%)		
	No smoker	34 (52%)		
Histology	Inflammatory pseudotumor	5 (8%)		
	Inflammatory granulomatouslesions	7 (11%)		
	AC	24 (37%)		
	SCC	29 (45%)		
NSCLC stage (AJCC 9th ed.)	I	12 (18%)		
	II	10 (19%)		
	III	17 (26%)		
	IV	14 (22%)		
FDG PET data				
SUV_max_			11.37	1.33–42.35
SUL			9.13	1.10–35.15
MTV			15.02	0.04–654.59
TLG			72.71	0.06–4693.75
Total CTCs/5 mL			5	0–50
Epithelial CTCs/5 mL			4.5	0–27
CTC data				
Mixed CTCs/5 mL			4	0–31
Mesenchymal CTCs/5 mL			0	0–10

### 3.2 FDG uptake and CTC features

The primary tumors of all 65 patients exhibited FDG uptake on PET/CT. The SUV_max_, SUL, MTV, and TLG of all primary foci were 9.43 (range 1.33–42.35), 8.68 (range 1.10–35.15), 58.85 (range 0.04–654.59), 147.03 (range 0.04–929.26), 333.4 (range 0.06–4694.75), and 1218.37 (range 0.06–456451.30), respectively. The SUV_max_, SUL, MTV, and TLG of the 12 benign lesions were 7.14 ± 5.21 (range 1.33–16.69), 5.68 ± 4.13 (range 1.10–13.13), 6.09 (range 0.05–19.23), and 25.81 (range 0.07–45.96), respectively. Fifty‐three patients with NSCLC lesions had SUV_max_, SUL, MTV, and TLG values of 10.77 (1.46–42.35), 8.36 (range 1.10–35.15), 65.42 (range 0.04–654.59), and 354.75 (range 0.06–4693.69), respectively.

In all patients, the total CTCs, epithelial CTCs, mixed CTCs, and mesenchymal CTCs were 5 (range 0–50) per 5 mL, 4.5 (range 0–27) per 5 mL, 4 (range 0–31) per 5 mL, and 0 (range 0–10) per 5 mL, respectively. Total CTCs, epithelial CTCs, mixed CTCs and mesenchymal CTCs in patients with benign lesions were 5.25 (range 0–11)/5 mL, 5.25 (range 0–11)/5 mL, 0 (range 0–0)/5 mL, and 0 (range 0–0)/5 mL, respectively. The total CTCs, epithelial CTCs, mixed CTCs, and mesenchymal CTCs in NSCLC patients were 8.5 (range 0–50)/5 mL, 3.5 (range 0–27)/5 mL, 4.5 (range 0–31)/5 mL, and 0 (range 0–10)/5 mL, respectively.

### 3.3 Differences in FDG metabolic parameters, CTC counts and subtypes between benign lung diseases and NSCLC

**FIGURE 1:**
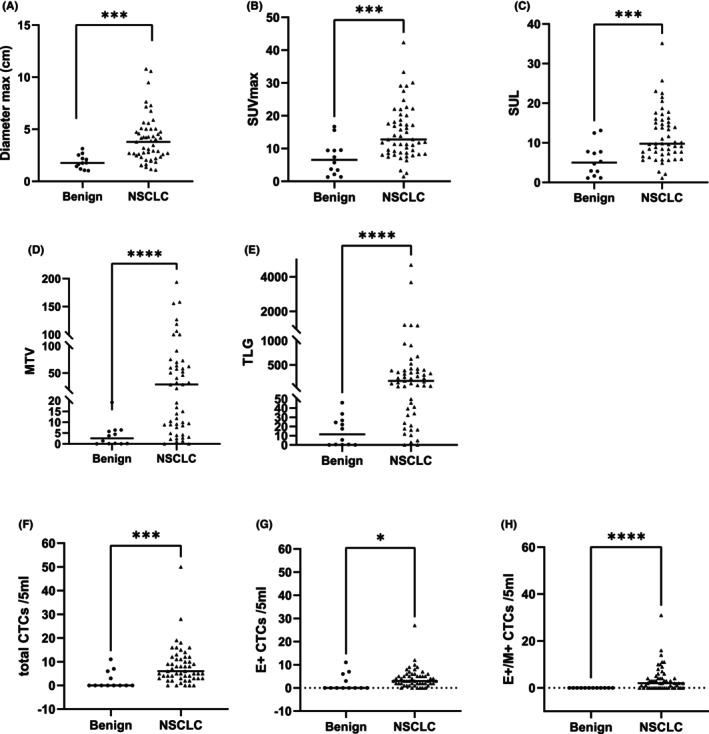
The differences between maximum diameter (A; *p* = 0.001), SUV_max_ (B; *p* = 0.001), SUL (C; *p* = 0.001), MTV (D; *p* < 0.001), TLG (E; *p* < 0.001), total CTCs (F; *p* = 0.001), epithelial CTCs (G; *p* = 0.04), and mixed CTCs (H; *p* < 0.001) by Mann–Whitney *U*‐nonparametric test in patients with benign lung diseases and NSCLC, respectively.*p < 0.05, ***p < 0.01. ****p < 0.001. E^+^ CTCs, Epithelial CTCs; E^+^/M^+^ CTCs, Mixed CTCs; NSCLC, Non‐small cell lung cancer.

Clinical data, FDG metabolic parameters, CTC number and CTC subtype characteristics were compared between the benign and malignant groups (Table 2). The statistical results revealed differences in the maximum diameter of the primary foci, SUV_max_, SUL, MTV, TLG, total CTCs, epithelial CTCs, and mixed CTCs between the two groups. The values in the NSCLC group were significantly greater than those in the benign lesion group (*p* < 0.05) (Figure 1). However, no significant differences were observed in sex, age, smoking history, or mesenchymal CTCs (*p* > 0.05).

**TABLE 2:** Benign lung occupations and NSCLC patients' characteristics.

	Benign (*N* = 12)	NSCLC (*N* = 53)	χ^2^/Z	*p*‐Value
Age	69 ± 3	70 ± 10	*T* = −1.756	0.087
Gender	Female 4 (33.3%)	Female 23 (43.4%)	χ^2^ = 0.408	0.523
	Male 8 (66.7%)	Male 30 (56.6%)		
Smoking	Yes 5 (41.7%)	Yes 26 (49.1%)	χ^2^ = 0.214	0.643
	No 7 (58.3%)	No 27 (50.9%)		
Diameter max	1.88 ± 0.19 (1.03–3.15)	2.61 (1.09–10.79)	*Z* = −3.720	0.001
SUV_max_	7.14 ± 1.50 (1.33–16.69)	10.77 (1.46–42.35)	*Z* = −3.213	0.001
SUL	5.68 ± 1.19 (1.10–13.13)	8.36 (1.10–35.15)	*Z* = −3.187	0.001
MTV	6.09 (0.05–19.23)	65.42 (0.04–654.59)	*Z* = −3.889	<0.001
TLG	25.81 (0.07–45.96)	354.75 (0.06–4693.75)	*Z* = −4.024	<0.001
Total CTCs	5 (0–11)	9 (0–50)	*Z* = −3.247	0.001
Epithelial CTCs	5 (0–11)	4 (0–27)	*Z* = −2.053	0.04
Mixed CTCs	0	5 (0–31)	*Z* = −3.745	<0.001
Mesenchymal CTCs	0	0 (0–10)	*Z* = −0.837	0.403

### 3.4 Differences in metabolic parameters, CTC counts and CTC subtypes between patients with benign lung diseases and patients with Stage I NSCLC

**FIGURE 2:**
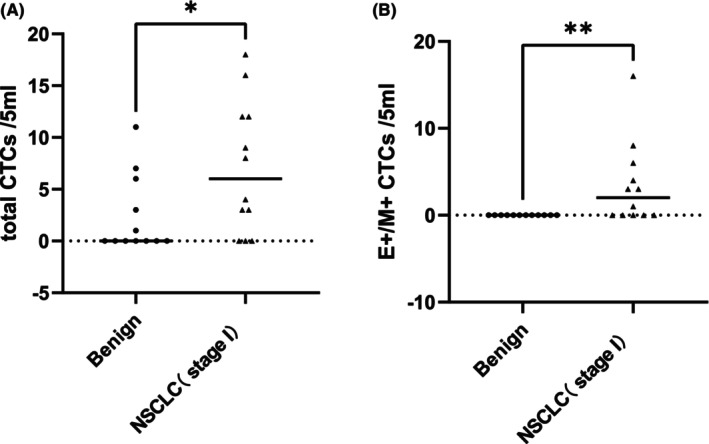
The differences between total CTCs (A; *Z* = −2.188, *p* = 0.039) and mixed CTCs (B; *Z* = −3.020, *p* = 0.014) by Mann–Whitney *U*‐nonparametric test in patients with benign lung diseases and Stage I NSCLC, respectively.*p < 0.05, **p < 0.01. NSCLC, non‐small cell lung cancer. E^+^/M^+^, epithelial and mesenchymal expressing.

Compared with benign lung diseases and Stage I NSCLC, only the total number of CTCs (*Z* = −2.188, *p* = 0.039) and the number of mixed CTCs (*Z* = −3.020, *p* = 0.014) differed between the two groups (Table 3). The values in the NSCLC group were significantly greater than those in the benign lesion group (*p* < 0.05) (Figure 2). There were no statistically significant differences in epithelial CTCs, mesenchymal CTCs, or FDG metabolic parameters between the groups.

**TABLE 3:** Benign lung occupations and I stage NSCLC patients' characteristics.

	Benign	NSCLC (Stage I)	χ^2^/t/Z	*p*‐Value
	*N* = 12	*N* = 12		
Age	69 ± 3	65 ± 2	*T* = −1.406	0.174
Gender	Female 4 (33.3%)	Female 4 (33.3%)	*χ* ^2^ = 0.000	1.000
	Male 8 (66.7%)	Male 8 (66.7%)		
Smoking	Yes 5 (41.7%)	Yes 6 (50.0%)	*χ* ^2^ = 0.168	0.682
	No 7 (58.3%)	No 6 (50.0%)		
Diameter max	1.88 ± 0.19 (1.03–3.15)	2.22 ± 0.27 (1.09–4.03)	*t* = −1.406	0.174
SUV_max_	7.14 ± 1.50 (1.33–16.69)	9.29 ± 1.55 (1.46–17.41)	*t* = −0.998	0.324
SUL	5.68 ± 1.19 (1.10–13.13)	7.09 ± 1.18 (1.10–13.78)	*t* = −0.843	0.409
MTV	6.09 (0.05–19.23)	11.33 (0.04–28.73)	*Z* = −0.693	0.514
TLG	25.81 (0.07–45.96)	54.59 (0.06–139.32)	*Z* = −0.693	0.514
Total CTCs	5 (0–11)	7 ± 2 (0–18)	*Z* = −2.188	0.039
Epithelial CTCs	5 (0–11)	4 ± 1 (0–8)	*Z* = −1.218	0.242
Mixed CTCs	0 (0–0)	2 (0–16)	*Z* = −3.020	0.014
Mesenchymal CTCs	0 (0–0)	0 (0–0)	*Z* = 0	1.0

### 3.5 Value of differential diagnosis

Using a tumor SUV_max_ of ≥2.5 as a diagnostic criterion and comparing it with pathology results, eight patients were misdiagnosed, with six benign lesions mistakenly identified as malignant and two Stage I NSCLC patients misdiagnosed as benign. 18F‐FDG PET/CT exhibited a sensitivity of 90.6% and a specificity of 50.0% for detecting NSCLC. In 12 Stage I patients, 10 patients were accurately diagnosed by 18F‐FDG PET/CT, and 2 patients were misdiagnosed. Among 41 Stage II–IV patients, all 41 were correctly diagnosed with no misdiagnoses. The sensitivity of PET/CT for detecting Stage I–IV NSCLC was 75.0%, 100.0%, 100.0%, and 100.0%, respectively.

Considering CTC≥1 per 5 mL as positive, 51 out of 65 patients (78.5%) were CTC positive, with 14 being CTC negative (8 benign lesions, 4 Stage I NSCLC, 2 Stage II NSCLC). The sensitivity of CTCs for detecting NSCLC was 78.5%, with a specificity of 57.1%. For Stage I–IV NSCLC, the sensitivities of CTCs were 66.7%, 80.0%, 100.0%, and 100.0%, respectively.

**FIGURE 3:**
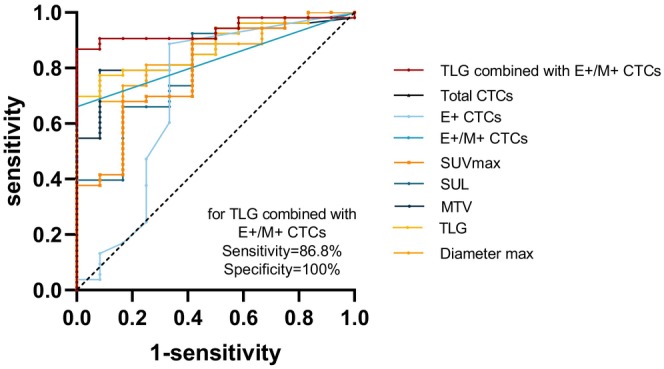
Receiver operating characteristic curve analyses in patients with NSCLC or benign disease. CTC, circulating tumor cell; E^+^, epithelial expressing; E^+^/M^+^, epithelial and mesenchymal expressing; M^+^, mesenchymal expressing; MTV, tumor volume of primary lesion; NSCLC, non‐small cell lung cancer; SUL, standard uptake value metabolic; SUV_max_, maximum standardized uptake value; TLG, total lesion glycolysis of primary lesion.

ROC curves were constructed based on the maximum diameter, FDG uptake parameters (SUV_max_, SUL, MTV, and TLG), and CTC characteristics (total CTCs, epithelial CTCs, and mixed CTCs) (Figure 3). The corresponding AUC values were 0.8459, 0.7987, 0.7964, 0.8616, 0.8742, 0.8003, 0.7068, and 0.8302, respectively. The critical values corresponding to the maximum Yoden index were 2.70, 7.43, 5.45, 7.04, 47.86, 0.5/5 mL, 1.5/5 mL, and 0.5/5 mL, respectively. Furthermore, combining the highest Jordon's index among CTCs (mixed CTCs) and metabolic parameters (TLG) increased the AUC [0.937; 95% CI, 0.879–0.995; *p* < 0.001] (Table 4). The sensitivity and specificity were 86.8% and 100.00%, respectively. The Delong test for two‐by‐two comparison of multiple ROC curves revealed that in the diagnosis of NSCLC with benign lung diseases, TLG combined with mixed CTCs was statistically different from all other diagnostic methods (*p* < 0.05) and higher than any other diagnostic criteria (Table 5).

**TABLE 4:** AUC value based on the maximum diameter, FDG uptake parameters (SUV_max_, SUL, MTV, TLG), and CTC characteristics.

	AUC value	*p*‐Value	95% CI
Epithelial CTCs	0.689	0.042	0.488–0.891
Mixed CTCs	0.830	0.000	0.733–0.928
Total CTCs	0.800	0.001	0.653–0.948
SUV_max_	0.799	0.001	0.663–0.935
SUL	0.796	0.001	0.660–0.933
MTV	0.862	0.000	0.766–0.957
TLG	0.874	0.000	0.789–0.960
Diameter max	0.846	0.000	0.745–0.946
TLG combined with mixed CTCs	0.937	0.000	0.879–0.995

**TABLE 5:** Comparison of the diagnostic efficacy of ROC curves for CTCs and its subtypes (total CTCs, epithelial CTCs, mixed CTCs), metabolic parameters (SUV_max_, SUL, MTV, TLG), and TLG combined with mixed CTCs.

	*z*	*p*‐Value	AUC differences	95% CI
Epithelial CTCs–MIXED CTCs	−1.121	0.262	−0.123	−0.339 to 0.092
Epithelial CTCs–Total CTCs	−2.333	0.02	−0.094	−0.172 to −0.015
Epithelial CTCs–SUV_max_	−1.031	0.303	−0.092	−0.267 to 0.083
Epithelial CTCs–SUL	−0.987	0.324	−0.09	−0.268 to 0.088
Epithelial CTCs–MTV	−1.605	0.109	−0.155	−0.344 to 0.034
Epithelial CTCs–TLG	−1.775	0.076	−0.167	−0.352 to 0.017
Epithelial CTCs–TLG combined with Mixed CTCs	−2.272	0.023	−0.23	−0.429 to −0.032
Epithelial CTCs Diameter max	−1.236	0.216	−0.139	−0.36 to 0.081
Mixed CTCs–Total CTCs	0.396	0.692	0.03	−0.118 to 0.178
Mixed CTCs–SUV_max_	0.403	0.687	0.031	−0.121 to 0.184
Mixed CTCs–SUL	0.437	0.662	0.034	−0.118 to 0.185
Mixed CTCs–MTV	−0.561	0.575	−0.031	−0.141 to 0.078
Mixed CTCs–TLG	−0.861	0.389	−0.044	−0.144 to 0.056
Mixed CTCs–TLG combined with Mixed CTCs	−3.157	0.002	−0.107	−0.173 to −0.041
Mixed CTCs–Diameter max	−0.273	0.785	−0.016	−0.129 to 0.097
Total CTCs–SUV_max_	0.024	0.981	0.002	−0.127 to 0.13
Total CTCs–SUL	0.059	0.953	0.004	−0.127 to 0.135
Total CTCs–MTV	−0.854	0.393	−0.061	−0.202 to 0.079
Total CTCs–TLG	−1.101	0.271	−0.074	−0.206 to 0.058
Total CTCs–TLG combined with Mixed CTCs	−1.969	0.04	−0.137	−0.273 to −0.001
Total CTCs–Diameter max	−0.517	0.605	−0.046	−0.218 to 0.127
SUV_max_–SUL	0.239	0.811	0.002	−0.017 to 0.022
SUV_max_–MTV	−0.897	0.37	−0.063	−0.2 to 0.075
SUV_max_–TLG	−1.239	0.215	−0.075	−0.195 to 0.044
SUV_max_–TLG combined with mixed CTCs	−2.133	0.033	−0.138	−0.266 to −0.011
SUV_max_–Diameter max	−0.542	0.588	−0.047	−0.218 to 0.123
SUL–MTV	−0.918	0.358	−0.065	−0.205 to 0.074
SUL–TLG	−1.255	0.21	−0.078	−0.199 to 0.044
SUL–TLG combined with mixed CTCs	−2.17	0.03	−0.141	−0.268 to −0.014
SUL–Diameter max	−0.561	0.575	−0.05	−0.223 to 0.124
MTV–TLG	−0.901	0.368	−0.013	−0.04 to 0.015
MTV–TLG combined with mixed CTCs	−2.154	0.031	−0.075	−0.144 to −0.007
MTV–Diameter max	0.523	0.601	0.016	−0.043 to 0.075
TLG–TLG combined with mixed CTCs	−2.188	0.029	−0.063	−0.119 to −0.007
TLG–Diameter max	0.818	0.414	0.028	−0.04 to 0.096
TLG combined with mixed CTCs–Diameter max	2.073	0.038	0.091	0.005 to −0.177

### 3.6 Differential diagnostic value of FDG metabolic parameters and CTC typing for benign lung occupancy and Stage I NSCLC

**FIGURE 4:**
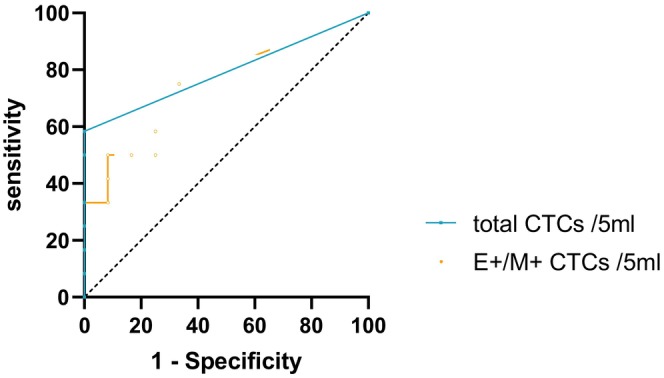
Receiver operating characteristic curve analyses in patients with Stage I NSCLC or benign disease. CTC, circulating tumor cell; E^+^/M^+^, epithelial and mesenchymal expressing; NSCLC, non‐small cell lung cancer.

ROC curves were generated based on the total CTCs and mixed CTCs between the benign lung occupancy and Stage I NSCLC groups, and the corresponding areas under the curve (AUCs) were 0.750 [95% confidence interval (CI), 0.600–0.983; *p* = 0.015] and 0.792 [95% confidence interval (CI), 0.551–0.949; *p* = 0.038], respectively (Figure 4). The critical values corresponding to the maximum values of the Jordon index were 3.5/5 mL and 2/5 mL, respectively. Comparing the difference in diagnostic efficacy of mixed CTCs and total CTCs for stage I NSCLC, there was no statistical difference between them (*p* = 0.480).

### 3.7 Differences in the total number and subtypes of CTCs in patients with different stages and pathological subtypes of NSCLC

**FIGURE 5:**
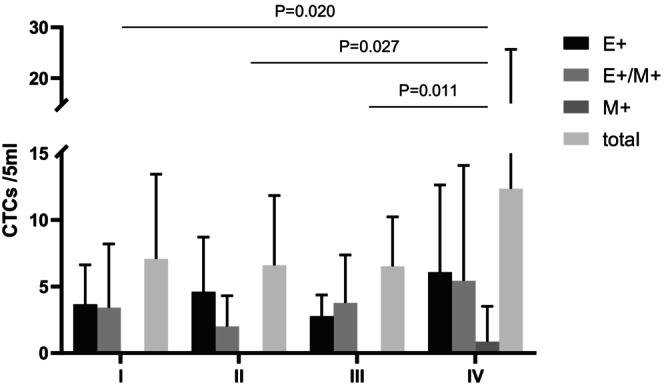
Differences in CTC characteristics in Stage I–IV NSCLC. Stage IV NSCLC exhibited a higher number of mesenchymal CTCs compared to those without distant metastases (Stage I–III) (*p* = 0.020, 0.027, and 0.011). E^+^, epithelial expressing; E^+^/M^+^, epithelial and mesenchymal expressing; M^+^, mesenchymal expressing; NSCLC, non‐small cell lung cancer.

**FIGURE 6:**
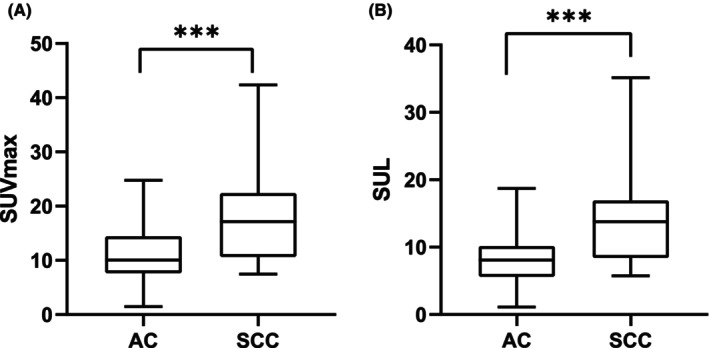
Differences in the SUV_max_ (A; *p* = 0.002) and SUL (B; *p* = 0.006) between patients with adenocarcinoma and squamous carcinoma. AC, adenocarcinoma; SCC, squamous carcinoma. ***p < 0.01.

We investigated the differences in CTC characteristics among patients with stage I‐IV NSCLC. The CTC detection rates of patients with Stage I–IV disease were 67%, 80%, 100% and 100%, respectively. There was no significant difference in the total CTCs or epithelial or mixed CTCs among NSCLC patients at different stages (*p* > 0.05), but there was a difference in mesenchymal CTCs. Further multiple comparisons indicated that individuals who progressed to distant metastases (Stage IV) exhibited a notably greater number of mesenchymal CTCs than did those without distant metastases (Stage I–III) (*p* = 0.020, 0.027, and 0.011, respectively) (Figure 5). Moreover, there were no differences in the total CTCS or each subtype between patients with squamous cell carcinoma and patients with adenocarcinoma. However, there was a notable difference in the SUV_max_ and SUL, with *p*‐values of 0.002 and 0.006, respectively, which were greater for squamous carcinoma than for adenocarcinoma (Figure 6).

### 3.8 Correlation of PET metabolic parameters and CTC subtypes in NSCLC

**FIGURE 7:**
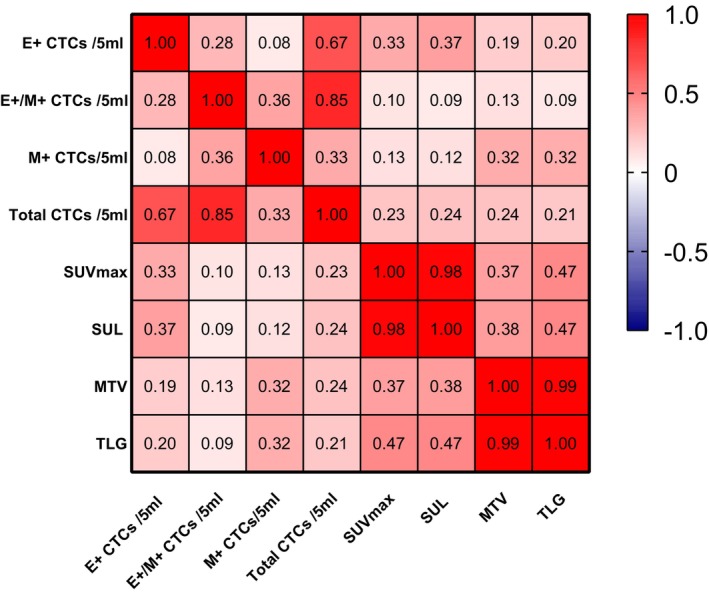
Heat map of correlation between FDG uptake parameters and CTC features. Red indicates positive correlation and blue indicates negative correlation. The number of epithelial CTCs correlated with SUV_max_ (*r* = 0.33, *p* = 0.015) and SUL (*r* = 0.37, *p* = 0.006). Mesenchymal CTCs exhibited correlations with MTV (*r* = 0.32, *p* = 0.018), and TLG (*r* = 0.32, *p* = 0.02). E^+^, epithelial expressing; E^+^/M^+^, epithelial and mesenchymal expressing; M^+^, mesenchymal expressing.

Spearman's correlation analysis was conducted for the metabolic parameters and CTCs of all NSCLC patients. The statistical results indicated that the number of epithelial CTCs correlated with the SUV_max_ (*r* = 0.33, *p* = 0.015) and SUL (*r* = 0.37, *p* = 0.006). Mesenchymal CTCs exhibited correlations with the MTV (*r* = 0.32, *p* = 0.018) and TLG (*r* = 0.32, *p* = 0.02) (Figure 7).

**FIGURE 8:**
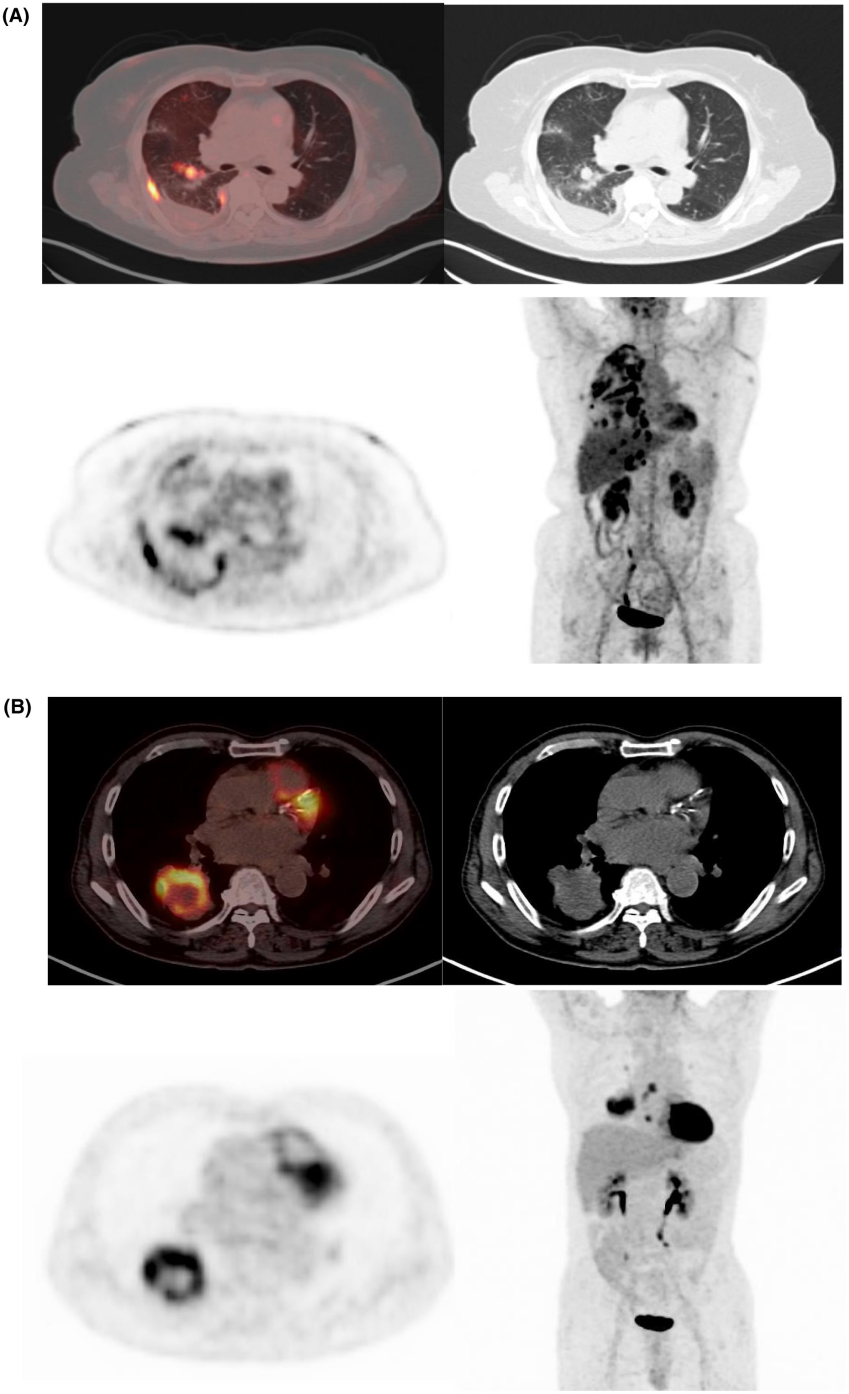
(A) Adenocarcinoma, Stage IVA, SUV_max_ was 5.46, SUL was 4.35, MTV was 7.77 cm^3^. A low MTV and TLG value of the primary site with pleural and mediastinal lymph node metastases occurred. The number of total CTCs was 20/5 mL, Epithelial CTCs was 9/5 mL, mixed CTCs was 10/5 mL and mesenchymal CTCs was 1/5 mL. (B) Squamous carcinoma, Stage IIIB, SUV_max_ was 11.32, SUL was 9.8, MTV was 50.54 cm^3^, TLG was 240.17 cm^3^, and central necrosis of the primary lesion resulted in reduced MTV and TLG. The number of total CTCs was 6/5 mL, Epithelial CTCs was 6/5 mL, mixed CTCs and mesenchymal CTCs was 0/5 mL.

All NSCLC patients were further classified into early stage (I–II) and intermediate‐late stage (III–IV) groups, and the correlation of FDG metabolic parameters with the number and subtypes of CTCs within each subgroup was analyzed. Spearman's correlation analysis revealed that epithelial CTCs were correlated with SUV_max_ (*r* = 0.543, *p* = 0.009) and SUL (*r* = 0.552, *p* = 0.008), and the total CTCs was correlated with SUV_max_ (*r* = 0.622, *p* = 0.003), SUL (*r* = 0.652, *p* = 0.003), MTV (*r* = 0.460, *p* = 0.031), and TLG (*r* = 0.472, *p* = 0.027) in the early group (Stage I–II) (Figure 8). Only mesenchymal CTCs was associated with MTV (*r* = 0.369, *p* = 0.041), and TLG (*r* = 0.415, *p* = 0.02) in the intermediate‐late group (Stage III–IV).

## 4 DISCUSSION

Early diagnosis and clinical staging of NSCLC are pivotal aspects of clinical practice. Several studies^15^, ^16^, ^17^, ^18^, ^19^, ^20^ have confirmed the distinctive role of CTC detection and 18F‐PET/CT imaging in diagnosing malignant tumors and identifying tumor metastasis. However, both methods have their own drawbacks. The reliance of FDG PET/CT on focal FDG uptake can lead to false‐negative results,^21^ particularly in cases such as small nodules, invasive mucinous adenocarcinomas with low cell counts, and low‐grade malignancies. Additionally, false‐positive outcomes^22^ (observed in inflammatory pseudotumors (43%), tuberculous tumors (37%), and histiocytic lung inflammation (6%)) are possible in differential diagnosis, necessitating cautious interpretation. Simultaneously, challenges exist in CTC detection and utilization: the scarcity of peripheral blood CTCs poses difficulties in capturing and isolating CTCs with high efficiency and purity from complex blood environments.^18^ Furthermore, CTCs undergo various adaptations and transformations, such as MET,^23^ to survive in novel and challenging environments. Since no single biomarker can universally identify all CTCs, relying on a single biomarker may lead to underestimation or false‐negative results. In this study, we detected CTCs utilizing multiple molecular probes and conducted subtype analysis. Additionally, we collected semi‐quantitative parameters from PET/CT scans that reflect tumor metabolism and load. This approach allowed for a more comprehensive exploration of the relationship between the two modalities in NSCLC, offering new insights into diagnostic methods and staging strategies for NSCLC.

The primary research objectives were achieved in this study. Initially, both FDG PET metabolic parameters and CTCs demonstrated diagnostic value for NSCLC, and combining TLG with mixed CTCs could enhance their diagnostic efficacy. Furthermore, the total CTCs and mixed CTCs showed greater diagnostic value than FDG PET in distinguishing benign lesions from stage I NSCLC. Notably, in NSCLC patients, epithelial CTCs exhibited a positive correlation with the SUV_max_ and SUL, reflecting the glycometabolic activity of primary lesions, while mesenchymal CTCs correlated with the MTV and TLG, indicating tumor burden. Besides, epithelial CTCs showed stronger correlations with SUV_max_ and SUL, and total CTCs showed stronger correlations with SUV_max_, SUL, MTV, and TLG in stage I‐II NSCLC. Only mesenchymal CTCs in Stage III–IV NSCLC showed correlations with MTV and TLG. Stage IV NSCLC patients with distant metastases displayed a greater number of mesenchymal‐type CTCs, whereas the total CTCs and epithelial and mixed CTCs did not significantly differ across Stages I–IV NSCLC.

In this study, we initially evaluated the sensitivity and specificity of SUV_max_ ≥2.5 and CTC ≥ 1/5 mL as diagnostic indicators of NSCLC. Our findings revealed that the sensitivity of both was high (90.6% vs. 78.5%); however, the specificity was not satisfactory (50.0% vs. 57.1%), consistent with prior research.^24^ This underscores the shortcomings of relying on SUV_max_ and unsubtyped CTC as standalone diagnostic tools for NSCLC. Further statistical analysis revealed differences in the maximum diameter, SUV_max_, SUL, MTV, TLG, total CTCs, epithelial CTCs, and mixed CTCs between the two groups (Figures 1 and 2), all of which were greater in the NSCLC group and held diagnostic value. Subsequently, the diagnostic efficacy of metabolic parameters and different subtypes of CTCs for NSCLC were analyzed separately. In order to demonstrate that the combination of the two methods can enhance diagnostic efficacy, we combined TLG and mixed CTCs, two indicators with high diagnostic efficacy, and analyzed whether the combined indicator differed from the others. The results showed that the combined index was statistically different from the other diagnostic indexes, and this combined index greatly increased the specificity (100%). In our study, among patients with benign lung diseases, four CTC‐positive patients were identified, all of whom displayed an epithelial phenotype. Previous studies^25^, ^26^ have noted that tumor markers such as CK and Epcam can be expressed in benign cells, potentially leading to false‐positive results and compromising specificity. In the identification of CTCs, as in most studies, our study used the biomarker CD45 to identify and exclude leukocytes. CD45 is a receptor protein tyrosine phosphatase that is expressed on the surface of all nucleated hematopoietic cells and their precursors.^27^ However, it has been shown that some leukocyte subpopulations express low levels of CD45 or do not express CD45, but nonspecific staining for cytokeratins, incorrectly identifying them as CTC, such as neutrophils,^28^ myeloid‐derived suppressor cells^29^ or other immature blastomyeloid cell populations.^30^ In addition, there is evidence that granulocyte subpopulations of leukocytes can stain non‐specifically for CK, especially neutrophils.^31^ These issues increase the risk of CTC misclassification after CTC enrichment. Researches have shown that using a combination of CD66b (a granulocyte marker),^32^ CD15 (another granulocyte marker),^28^ and CD11b (a bone marrow marker),^33^ along with CD45 for double staining can clearly differentiate white blood cells from CTC, which present with CK^+^ staining. Thus reducing false positive rates. Additionally, some studies^34^, ^35^ have used immunomagnetic beads to enrich white blood cells expressing CD45 and CD15 from blood samples to capture CTC. This detection method can identify non‐epithelial tumor cells and those that have undergone EMT and lost detectable EpCAM expression, making it applicable to a wide range of cancer types. In future studies, it may be possible to be able to reduce the incidence of such false‐positive events by developing more specific isolation techniques and biomarkers. Besides, the development of epithelial‐type tumor markers specific to NSCLC could enhance assay specificity. In contrast to previous findings,^14^, ^36^ the detection rate of mesenchymal CTCs in this study was low (found in only 4 Stage IV NSCLC patients), limiting their diagnostic value in NSCLC, possibly due to sample size bias and the small number of advanced NSCLC patients. Mixed CTCs have emerged as the most effective subtype for differentiating benign and malignant conditions, consistent with prior research.^36^ Additionally, among the FDG uptake parameters, TLG displayed superior diagnostic efficacy. Meanwhile, the diagnostic efficacy can be further improved by combining TLG with mixed CTCs, with a sensitivity and specificity of 86.8% and 100.00%. PET/CT and CTCs provide more valuable information for the diagnosis of NSCLC. Although the majority of nodules can be diagnosed based on the CT findings, the presentations are diverse and lack specificity. There is considerable overlap in the CT morphology of benign and malignant lesions. A number of the signs that are considered to be of critical importance for the detection of lung cancer also bear resemblance to certain benign conditions. Consequently, misdiagnosis of solitary pulmonary nodule using CT is common, as distinguishing benign lesions based solely on morphology is challenging.^37^ Most of the cases included in this study had undergone CT screening, and some lung nodules (especially Stage I–II NSCLC) had atypical CT signs, making them challenging cases even for experienced radiologists, who found it hard to suggest a definitive diagnosis. In this instance, PET and CTC can provide additional diagnostic information. In short, while HRCT is usually a strong tool for diagnosing and assessing NSCLC, PET and CTC testing can also offer valuable supplementary insights, particularly in early‐stage NSCLC.

Additionally, we compared FDG metabolic parameters and CTC subtypes between the benign lung diseases group and the Stage I NSCLC group. The findings revealed no difference in FDG metabolic parameters between the two groups, suggesting that FDG uptake parameters alone may not distinguish between benign lung diseases and early‐stage lung cancer. Regarding CTC phenotypes, although the number of epithelial CTCs did not significantly differ, mixed CTCs were not detected in the benign lung diseases group. The total CTCs and mixed CTCs varied between the two groups, with higher levels observed in the Stage I NSCLC group than in the benign diseases group, consistent with previous research.^14^ The total CTCs and mixed CTCs can compensate for the limitations of FDG PET in differentiating benign conditions and Stage I NSCLC, offering valuable diagnostic insights. Similar sensitivities were observed for both methods in intermediate and advanced stage of NSCLC, consistent with prior studies.^24^

This study also examined CTCs and their subtypes characteristics across various stages of NSCLC. The findings indicated the highest CTC detection rate in intermediate and advanced NSCLC stages, but there was no significant difference in total CTCs, epithelial CTCs, or mixed CTCs among different NSCLC stages. The established EMT‐MET tumor metastasis theory^38^ suggests a dynamic shift in the cellular phenotype during tumor development and metastasis. EMT involves the transition of tumor cells from epithelial to mesenchymal characteristics and is crucial for primary tumor evolution and progression. It initiates early in disease progression, displaying diverse statuses during metastatic dissemination. Conversely, MET reverses EMT, resulting in epithelial features such as EpCAM or ECAD re‐expression at metastatic sites. Hence, various CTC types coexist throughout tumor progression,^39^ and analyzing the percentage of mixed CTCs with epithelial and mesenchymal expression may reveal differences in mixed CTCs at distinct stages.

The study acknowledges potential statistical errors due to the limited sample size. Moreover, mesenchymal CTCs were identified in a minority of Stage IV patients, with a significant difference between the nondistant metastatic (Stage I–III NSCLC patients) and distant metastatic (Stage IV NSCLC patients) groups. This finding suggests that elevated mesenchymal CTC levels are correlated with an increased risk of distant metastasis, underscoring the significant role of EMT in NSCLC spread and its association with poor prognosis in cancer patients.^40^, ^41^

Some studies^9^, ^10^ have molecularly confirmed the close association between EMT and reprogrammed cancer metabolism. EMT involves a dynamic shift in epithelial cell polarity and the adoption of spindle‐shaped, migratory mesenchymal characteristics, which are crucial for invasion and migration. Reprogrammed metabolism, exemplified by the Warburg effect, is another hallmark of cancer progression and is characterized by increased glucose uptake and lactate secretion even under oxygen‐rich conditions. The accelerated tumor division triggering metastatic dissemination may manifest as increased FDG uptake in FDG PET scans.^42^ Recent studies have explored the link between 18F‐FDG PET/CT metabolic parameters and CTCs in lung cancer patients, yielding varied findings. Bian et al.^43^ reported no significant correlation between CTCs and the SUV_max_ in stage IIB‐IVB NSCLC patients. Lei et al.^44^ found no association between baseline CTC count and WBMTV or disease stage. Conversely, Avella et al.^45^ observed a positive correlation between CTC count and both MTV and TLG. Castello et al.^46^ noted significant correlations between changes in CTC count during ICI treatment and baseline MTV and TLG, suggesting that CTCs in peripheral blood may mirror tumor burden. The discrepancies in the results could stem from differences in CTC detection methods, tumor biology, sample differences, and patient numbers. In our study of stage I‐IV NSCLC patients, a correlation analysis of metabolic parameters and CTCs revealed a positive correlation between epithelial CTCs and the SUV_max_/SUL, reflecting the glycometabolic activity of primary lesions. The number of mesenchymal CTCs correlated with the MTV and TLG, which are indicative of tumor burden. These findings imply that CTCs offer insights into metabolic activity and tumor burden, potentially serving as markers of aggressiveness in advanced NSCLC. In our study, the results of the correlation analysis conducted demonstrated that epithelial CTCs exhibited a correlation with SUV_max_ and the SUL, while mesenchymal CTCs demonstrated a correlation with MTV. However, the strength of the correlation was not significant. In the NSCLC group of patients with all clinical stages, there was a large heterogeneity of samples, which may have interfered with the analysis of FDG uptake and CTC correlation. To further explore this issue, we classified the NSCLC patients into early stage (Stage I–II) and intermediate‐late stage (Stage III–IV) groups, and analyzed the correlation of FDG metabolic parameters with CTCs within each subgroup. Statistical results showed that metabolic parameters in early‐stage NSCLC exhibited a stronger correlation with CTC and its subtypes. It may due to the fact that, on the one hand, some of the patients with Stage III–IV NSCLC have small foci but large metastases, and calculating the MTV and TLG of the primary foci alone is insufficient to account for the systemic tumor load (Figure 8A). On the other hand, some of the primary foci were large resulting in internal liquefaction necrosis, which led to low MTV and TLG values when compared with those without necrosis (Figure 8B). It is difficult to reflect the overall metabolic situation by only calculating the MTV and TLG of the primary lesion. Whole body MTV and whole body TLGW represent the systemic tumor load of the entire body, including the primary tumor, local lymph nodes and all distant metastases, can represent the overall metabolic characteristics of the tumor lesion better^47^ and may be able to better reflect the relationship between CTC and metabolic parameters. In addition, there is significant heterogeneity between tumor cells in primary tumors and metastatic sites, and it has been shown that there is a significant correlation between the results of liquid biopsy and tumor metabolism in metastatic lesions rather than primary tumors.^48^ This suggests that the biological behavior of tumors represented by the two kinds of biomarkers. Therefore, metabolic parameters and CTCs reflect the different biological characteristics of NSCLC. They cannot replace each other and are combined together to reveal the tumor landscape more completely.

In conclusion, our study involved patients with benign lung diseases and Stage I–IV NSCLC who underwent pretreatment with 18F‐FDG PET/CT and CTC analysis. Two key clinical implications emerged: first, both FDG PET metabolic parameters and CTCs hold diagnostic value for NSCLC, with PET demonstrating superior diagnostic efficacy, further enhanced by combining TLG and mixed CTCs. Second, total CTCs and mixed CTCs proved more valuable than FDG PET in distinguishing benign conditions from Stage I NSCLC. Notably, in NSCLC patients, epithelial CTCs correlated positively with the SUV_max_ and SUL, reflecting glycometabolic activity, while mesenchymal CTCs correlated with the MTV and TLG, reflecting tumor burden. Besides, epithelial CTCs showed stronger correlations with SUV_max_ and SUL, and total CTCs showed stronger correlations with SUV_max_, SUL, MTV, and TLG in Stage I–II NSCLC. Only mesenchymal CTCs in Stage III–IV NSCLC showed correlations with MTV and TLG. Mesenchymal CTCs serve as biomarkers for distant metastasis in NSCLC. It should be noted that the above metabolic parameters generally correlate with CTC subtypes, and further studies are needed to demonstrate a causal relationship.

However, our study has limitations, including its small sample size. Additionally, the PCR‐based method used, while sensitive and reproducible, may yield false‐positive signals in noncancerous cells. Future research should explore new CTC detection platforms. Finally, our study lacked posttreatment follow‐up data to assess the prognostic value of CTCs and FDG PET in NSCLC patients.

## 5 CONCLUSION

Both FDG PET metabolic parameters and CTCs have diagnostic value for NSCLC, and combining TLG with mixed CTCs could enhance the diagnostic efficacy. Total CTCs and mixed CTCs are more valuable than FDG PET in differentiating benign conditions from stage I NSCLC. Epithelial and mesenchymal CTC levels in peripheral blood correlate with tumor glucose metabolism in NSCLC patients. Mesenchymal CTCs can serve as biomarkers for distant metastasis in NSCLC.

## AUTHOR CONTRIBUTIONS

**Momo Sun:** Conceptualization (lead); formal analysis (lead); investigation (lead); methodology (lead); software (lead); writing – original draft (lead). **Dongyan Lu:** Investigation (equal); methodology (equal); software (equal). **Xiaoping Li:** Resources (equal); supervision (equal). **Jin Wang:** Investigation (equal); methodology (equal); software (equal). **Liang Zhang:** Resources (equal); supervision (equal). **Pan Yang:** Resources (equal); validation (equal). **Yang Yang:** Investigation (equal); software (equal). **Jie Shen:** Conceptualization (lead); funding acquisition (lead); project administration (lead); supervision (lead); writing – review and editing (lead).

## FUNDING INFORMATION

This study was supported by study on nuclide nanoprobes with aggregation‐induced luminescence properties in the integration of diagnosis and treatment of atherosclerotic vulnerable plaque in rabbits (21JCYBJC01060).

## CONFLICT OF INTEREST STATEMENT

The authors declare that they have No competing financial interests exist.

## CONSENT

All authors approved the final manuscript and the submission to this journal.

## Data Availability

The datasets generated during and/or analyzed during the current study are not publicly available, but are available from the corresponding author on reasonable request.
